# Safety of Delivering Eating Disorders Day Treatment Programme on the Virtual Platform in (COVID-19) Pandemic

**DOI:** 10.1192/bjo.2022.91

**Published:** 2022-06-20

**Authors:** Adaora Obiekezie, Claudia Friel, Mohammad Tayeem Pathan

**Affiliations:** Surrey and Borders Partnership NHS Foundation Trust, London, United Kingdom

## Abstract

**Aims:**

Intensive treatment for eating disorders include day treatment programme and specialist inpatient. COVID-19 pandemic led to lockdown in the UK on the 23rd March 2020. Adult Eating Disorders Day Treatment Programme in Surrey started delivering their care on the virtual platform from that date. It offered a combination of ‘virtual’ only and ‘blended’ care (virtual and in person) for more than a year. This service evaluation examined the safety of delivering intensive eating disorders treatment on the virtual platform.

**Methods:**

Data from March 2020 to March 2021 were retrospectively collected from Electronic patient record. Two clinicians collected the data on age, referral origin, accommodation, employment status, diagnosis (subtype), length of illness, comorbid mental and physical health diagnosis, duration of day care treatment, medication, admission weight and BMI, discharge weight and BMI, changes in bloods and ECG, acute hospital admission, risk-to-self events, admissions to Specialist Eating Disorders Unit and reasons for discharge.

**Results:**

Data indicated that 21 patients were admitted in day treatment programme over 1 year period. 10 patients had solely virtual treatment and 11 patients had blended day treatment programme. 11 patients had anorexia nervosa restrictive subtype, 5 patients had Anorexia Binge purge subtype and 5 patients had Anorexia Nervosa, Unspecified.

Average length of illness was 4.49 years. Mean age for the group was 24.7 years and most patients lived with family (n 18) and were unemployed (n 11). More than 2/3rd (76%) patients had comorbid mental health diagnosis and 48% (n 10) had comorbid physical health diagnosis.

Average length of admission was 5.26 months. Mean BMI on admission was 15.3 (Range 12–19) and mean BMI on discharge was 16.9 (Range 13.65–22).

Safety and outcome data indicated that there were no serious incidents recorded in that time period. 1 (5%) patients required admission to acute hospital as their physical health deteriorated. 8 (38%) patients required specialist inpatient admission as the day care did not affect any changes to their eating behaviours, and 4 (19%) patients had events indicating self harm episodes(19%).

**Conclusion:**

Our service evaluation data indicated that it is relatively safe to deliver day treatment programme on the virtual platform. Weekly face to face physical health monitoring (weight, BP, Pulse, temperature) and regular physical health investigations (Blood tests and ECG) were integral part of managing risks to health. On the other hand, delivering day treatment programme on the virtual platform has enabled the day treatment programme to prevent any significant outbreak of COVID-19 in a vulnerable group of patients and allowed them to receive uninterrupted support during pandemic.

